# Dermoscopy – a simple and rapid *in vivo* diagnostic technique for tinea incognito^☆^^☆☆^

**DOI:** 10.1016/j.abd.2019.09.017

**Published:** 2019-09-30

**Authors:** Sidharth Sonthalia, Balachandra S. Ankad, Mohamad Goldust, Abhijeet Kumar Jha

**Affiliations:** a Department of Dermatology, Venereology, and Trichology, SKINNOCENCE: The Skin Clinic & Research Center, Gurugram, India; b Department of Dermatology & STD, S. Nijalingappa Medical College, Bagalkot, India; c Department of Dermatology & STD, Mazandaran University of Medical Sciences, Sari, Iran; d Department of Dermatology & STD, Patna Medical College & Hospital, Patna, India

**Keywords:** Dermoscopy, Hair, Tinea, Weights and measures, Dermatoscopy, Corticosteroid abuse, Dermatophytosis, Cruris, Corporis, Tinea of vellus hair, Morse-code hairs, Bar-code hairs, Comma-shaped hairs, Corkscrew-shaped hairs, Black dots, Broken hairs, Deformable hairs, Translucent hairs

## Abstract

Tinea incognito resulting from corticosteroid abuse is becoming very common in the tropics. Its diagnosis is tricky owing to its confusing morphology, as well as practical and technical issues associated with mycological tests. Dermoscopy has now evolved as a novel diagnostic tool for diagnosing tinea incognito in such challenging situations, since the typical hair changes such as Morse-code hairs, deformable hairs, translucent hairs, comma and cork screw hairs, and perifollicular scaling may be seen despite steroid use, irrespective of mycological results.

Topical corticosteroid abuse not only renders therapeutic management challenging, it is contributing to the growing epidemic of antifungal therapeutic failure.^1^, ^2^ The utility of dermoscopy in rapid diagnosis of tinea capitis is well-established.^3^ However, dermoscopic diagnosis of tinea corporis, especially the incognito variant, has been sparingly reported.^4^

A 22-year-old medical undergraduate student presented with four-month-old itchy pinkish-red lesions in the right axilla. The lesion had ill-defined borders, and a shiny surface with peripherally scattered, mildly scaly papules (Fig. 1). He had been self-medicating with clobetasol-miconazole cream and oral itraconazole 200 mg/day, intermittently. Polarized dermoscopy revealed patchy erythema, perifollicular scales and casts, black dots, broken hairs, bent deformable hairs, Morse-code hairs, comma and cork-screw hairs, and translucent hairs; additionally, dotted vessels and telangiectasias were present (Fig. 2). These dermoscopic changes typify the tinea of non-glabrous skin.^2^, ^3^, ^4^, ^5^ Skin scrapings were sent for fungal culture and showed septate branching hyphae on 10% KOH microscopy. Oral terbinafine 250 mg/day and topical ciclopirox olamine 1% cream for six weeks resulted in complete resolution. *Trichophyton mentagrophytes var. interdigitale* was confirmed on culture.

**Figure 1:**
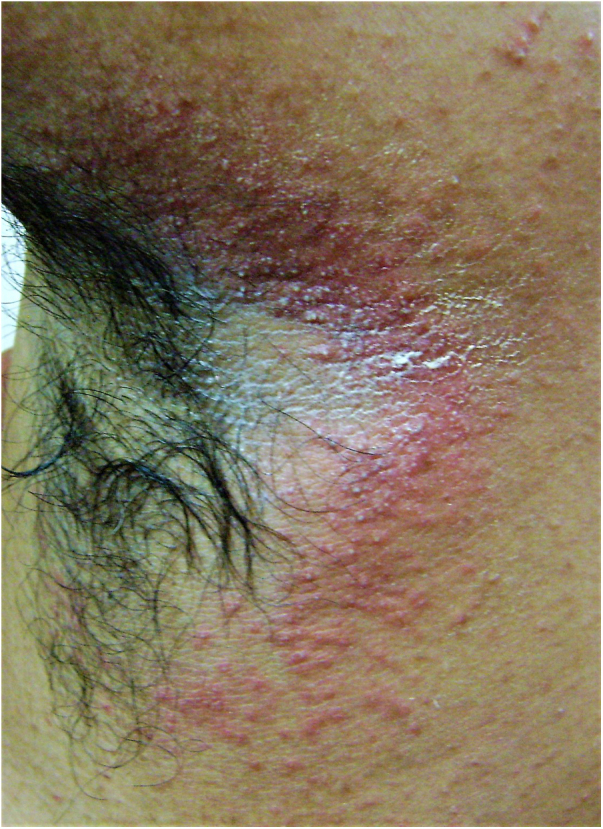
Clinical image of tinea incognito lesion over the right axilla of a young male – minimally raised erythematous plaque with ill-defined borders, shiny surface with peripherally scattered, mildly scaly papules. Onset four months previously; history of intermittent application of steroid-antifungal cream and oral itraconazole intake.

**Figure 2:**
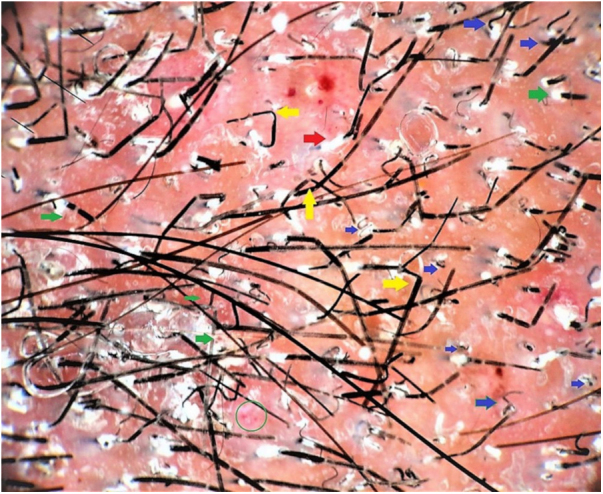
Polarized dermoscopic image of the lesion revealed patchy erythema, perifollicular scales (green arrow), and casts (red arrow), black dots, broken hairs, and comma and cork-screw hairs (blue arrows). The entire field is filled with translucent and deformable hairs with bends (yellow arrows), and Morse-code hairs showing horizontal skip white bands. Additionally, dotted vessels (green circle) and scattered telangiectasias (green arrows) were seen. The larger red blotches represent excoriation-induced, dried up blood-crusts (Dermlite 4, ×20).

Altered morphology, logistic issues associated with in-house light-based microscopy, and time delay of fungal culture results warrant a rapid office tool like dermoscopy to diagnose tinea incognito.^1^, ^2^ Fungal invasion of the hair leads to deformation and cracking that present as translucent, deformable hairs, comma and cork screw hairs, and Morse-code hairs that show horizontal skip white bands (localized invasion).^3^, ^4^, ^5^ Dermoscopy serves as a noninvasive and simple method that allows speedy *in vivo* diagnosis of tinea incognito.

## Funding

None declared.

## Author's contribution

*Sidharth Sonthalia*: Approval of the final version of the manuscript; conception and planning of the study; elaboration and writing of the manuscript; obtaining, analyzing and interpreting the data; intellectual participation in propaedeutic and/or therapeutic conduct of the cases studied; critical review of the literature; critical review of the manuscript.

*Balachandra S. Ankad*: Approval of the final version of the manuscript; elaboration and writing of the manuscript; critical review of the literature; critical review of the manuscript.

*Mohamad Goldust*: Approval of the final version of the manuscript; critical review of the literature; critical review of the manuscript.

*Abhijeet Kumar Jha*: Approval of the final version of the manuscript; critical review of the manuscript.

## Conflicts of interest

None declared.
